# Institutional analysis of stereotactic body radiotherapy (SBRT) for oligometastatic lymph node metastases

**DOI:** 10.1186/s13014-017-0820-1

**Published:** 2017-06-21

**Authors:** Rosanna Yeung, Jeremy Hamm, Mitchell Liu, Devin Schellenberg

**Affiliations:** 1 Department of Radiation Oncology, British Columbia Cancer Agency- Vancouver Center, 600 West 10th Avenue, Vancouver, BC V5Z 4E6 Canada; 2 Cancer Surveillance and Outcomes, British Columbia Cancer Agency, 703-686 West Broadway, Vancouver, BC V5Z 4C1 Canada

**Keywords:** Oligometastatic, Lymph nodes, Stereotactic body radiotherapy

## Abstract

**Background:**

In limited metastatic burden of disease, stereotactic body radiotherapy (SBRT) has been shown to achieve high local control rates. It has been hypothesized that SBRT may translate to a better quality of life by delaying the need for systemic chemotherapy and possibly increasing survival. There is limited published literature on the efficacy of SBRT in limited nodal metastases. The primary aim is to review institutional outcomes of patients with solitary or oligometastatic lymph nodes treated with SBRT.

**Methods:**

A retrospective study of patients treated with SBRT to metastatic lymph nodes (March 2010–June 2015) was conducted. Endpoints of this study were local control (LC), chemotherapy-free survival (CFS) following SBRT, toxicities, progression free survival (PFS), and overall survival (OS).

**Results:**

Eighteen patients with a mean age of 65 years underwent SBRT to metastatic lymph nodes. Median follow-up was 33.6 months. There were four hepatocellular carcinoma, seven colorectal, four pancreatic, one esophageal, one gallbladder and one lung primary. Eleven (61%) patients had lymph node metastases at initial presentation of metastatic disease. Seven patients (39%) had systemic therapy prior to SBRT, with five patients receiving two lines of chemotherapy. Eight patients had solitary metastatic disease at the time of radiotherapy. All patients had <5 metastases. Median size of lymph node metastases was 1.95 cm (range: 0.8–6.2 cm). RT doses were 31 to 60 Gy in four to ten fractions, with 44% of patients receiving 35 Gy in 5 fractions. At 1 year, LC was 94% and CFS from SBRT was 60%. One-year PFS and OS were 39% and 89% respectively. There were no grade 3 or higher toxicities.

**Conclusions:**

In this single institution study, SBRT to oligometastatic lymph nodes provided excellent LC and a moderate chemotherapy-free interval with minimal toxicities. Disease progression remains prominent in these patients and larger studies are warranted to identify those who benefit most from SBRT.

## Introduction

The concept of metastatic disease in cancer treatment has evolved over the past decades. Historically, the presence of metastases has been regarded as a clinical manifestation of widespread microscopic disseminated disease. From this historical perspective, local treatment to metastatic lesions cannot eradicate all cancer cells, and similarly, systemic therapies although prolonging survival, cannot offer a cure [1, 2].

In 1995, the concept of oligometastases was introduced as a distinct clinical entity of limited metastatic disease; this is an intermediate stage of cancer spread between localized and disseminated disease [1]. Local control of limited metastatic disease is believed to improve systemic control and achieve potential cure as it is hypothesized that the cancer has not yet acquired the genetic variation required for widespread dissemination [1–3]. Furthermore, it has been hypothesized that local treatment of limited metastases may delay the need for systemic therapies, thereby improving quality of life in the short term and providing more lines of cytotoxic therapy in the long term [4].

Local cancer treatments such as surgical metastasectomy have confirmed the distinct biological entity of oligometastases. Many published reports have demonstrated improved survival and long term disease control after surgical resection of metastases in selected cancer histologies [5–9]. Stereotactic body radiotherapy (SBRT) has emerged as another local treatment option for oligometastases with local control rates reported up to 90% at 2 years [10, 11]. Most data for SBRT in treatment of oligometastases, however, pertains to treatment of liver and lung metastases [10, 11]. There is limited published literature on the efficacy of SBRT in limited nodal metastases [11, 12].

The primary objective of this study is to report the clinical outcomes in a series of patients with limited lymph node metastases treated with SBRT at our institution. Study endpoints were local control (LC), chemotherapy-free survival (CFS) following SBRT, toxicities, progression free survival (PFS), and overall survival (OS).

## Methods and materials

### Patient population

This is a retrospective study of patients treated with SBRT on an institutional protocol to metastatic lymph nodes between March 2010 and June 2015 at the British Columbia Cancer Agency in Canada. Patients were included if they had fewer than or equal to five metastases at the time of SBRT with at least one treated site being a lymph node. Although there are variable definitions of oligometastatic disease, fewer than or equal to five metastases was used in this study, as this is consistent with the definition used in many published series and a recent international clinical trial [13–15]. The diagnosis of oligometastatic disease was based on clinical examination and imaging studies, preferably by positron emission tomography/computed tomography (PET/CT) scan although imaging with CT alone or MRI was permitted. Patients were included in the analysis if they were ≥ 18 years old, had an Eastern Cooperative Oncology Group (ECOG) score ≤ 2, have had a complete primary tumor response, and their lymph node metastases deemed unresectable by multidisciplinary tumor boards or from a formal surgical consultation. Patients were excluded from the analysis if they have been previously treated with SBRT to the same site or had documented intracranial metastases. Approval for this study was obtained from the British Columbia Cancer Agency Research Ethics Board.

### Treatment and follow-up

Patients were simulated by CT scan in a supine position with arms above their head and immobilized with a vacuum bag. Additional thermoplastic upper body or head and neck masks were used for treatment of upper thoracic and cervical regions. No fiducials were placed with the exception of treating portahepatic or periportal regions. For these patients, 3–5 fiducial markers were implanted into the parenchyma of the liver under ultrasound guidance close to the porta hepatis, one to two weeks prior to simulation.

A standard CT scan with contrast was obtained for all patients at simulation. An additional 4DCT scan was obtained for lymph nodes that were felt to be mobile with respiration at the discretion of the treating radiation oncologist. Baseline diagnostic PET/CT scans, if available, were fused with the planning CT scans to aid in tumor delineation. Gross tumor volume (GTV) was defined as disease seen on the CT simulation scan and/or the PET/CT scan. The clinical target volume (CTV) was kept equivalent to the GTV. In the cases with 4DCT simulation, an internal target volume (ITV) was the GTV seen on all phases of the 4DCT scan. Planning target volume (PTV) corresponded to the ITV with a 3–5 mm isotropic margin. For patients who did not undergo 4DCT simulation scan, an isotropic margin of 5–10 mm on the CTV was used to derive the PTV. PTV margins were chosen according to the accuracy of the fusion between the diagnostic and planning CT scans as well as the visibility of the target lesion on the planning CT.

All patients were treated with a VMAT plan, with the exception of one patient who was treated with IMRT. Treatment was delivered using 6 MV photons. The dose prescribed to the PTV ranged from 31 to 60 Gy in four to ten fractions. Treatment doses were individualized based on location of tumor in relation to tolerance of nearby organs at risk. The aim was to cover the PTV by 95% of the prescribed dose, but coverage of the PTV was placed as the lowest priority (below dose tolerances of organs at risk), and accepted if it was undercovered [16]. Daily image guidance with cone-beam CT scan was performed to localize the target before treatment delivery.

Patients were evaluated by radiation oncologists every 2 to 4 months after treatment. Routine follow-up imaging by PET/CT, CT or MRI was performed.

### Endpoints

Response Evaluation Criteria in Solid Tumors (RECIST) was used to assess local tumor response [17]. Failure of the local control endpoint was progression or recurrence within the PTV. Chemotherapy-free survival following SBRT was defined as the interval between the last day of SBRT and date of chemotherapy initiation or last follow-up for those not requiring chemotherapy. Toxicities from radiotherapy were graded according to the Common Terminology Criteria for Adverse Events (CTCAE) v 4.0. They were measured from the last day of SBRT and censored at the time of disease progression or subsequent therapy. Progression was defined as local failure (occurring within the PTV), regional failure or metastatic progression. Failure for the OS endpoint was death due to any cause and was measured from the last day of SBRT to the date of death or last follow-up.

Quantitative variables were reported by median and range, and qualitative variables by frequency and percentage. Results were calculated with SAS Version 9.3 for Microsoft Windows (SAS Institute Inc., Cary, NC). LC, CFS, PFS, and OS were analyzed using Kaplan-Meier survival analyses..

## Results

### Patient characteristics

Between March 2010 and June 2015, 18 patients with a mean age of 65 years (range: 31–88 years) underwent SBRT to a metastatic lymph node. Median follow-up was 33.6 months. One patient was ECOG 4, but was included in our study as his performance status was related to paraplegia from a remote motor vehicle accident unrelated to his cancer diagnosis. Baseline demographics are presented in Table 1.

**Table 1:** Patient demographics

Characteristics	All Patients (*N* = 18) n (%)
Age at SBRT, y (median (range))	65 (31, 88)
Gender (male/female)	11 (61.1)/7 (38.9)
ECOG status	
ECOG ≤ 1	17 (94.4)
ECOG ≥ 2	1 (5.6)
Primary diagnosis	
Liver	4 (22.2)
Colorectal	7 (38.9)
Esophagus	1 (5.6)
Pancreas	4 (22.2)
Gallbladder	1 (5.6)
Lung	1 (5.6)
Imaging for staging	
PET	16 (88.9)
CT	1 (5.6)
MRI	1 (5.6)
Number of lines of chemotherapy prior to SBRT	
0	11 (61.1)
1	2 (11.2)
2	5 (27.8)
Number of metastasis at time of SBRT	
1	8 (44.4)
2	6 (33.3)
3	1 (5.6)
4	3 (16.7)
SBRT treatment site	
Thorax	7 (38.9)
Abdomen	8 (44.4)
Pelvis	3 (16.7)

The most common primary tumor was colorectal cancer, accounting for more than a third of the patients. All but one patient (lung) had a gastrointestinal primary. Eight patients presented with abdominal lymph node metastases, seven with thoracic lymph nodes and three with pelvic lymph nodes. Eleven patients had lymph node metastases as part of their initial presentation of metastatic disease (range: 1 to 34 months from initial diagnosis), while seven patients had other initial sites of metastases (range: 10 to 64 months from original metastases to lymph node metastases). Median time from cancer diagnosis to first metastases was 16.5 months (range: 0 to 102 months). At the time of SBRT, eight patients (44%) had solitary metastatic disease, with all patients having four or fewer total sites of metastases.

### Treatment characteristics

Seven patients (39%) had systemic therapy prior to SBRT, with five of those patients (71%) receiving two lines of chemotherapy. No patients received more than 2 lines of chemotherapy prior to SBRT. For patients who received prior chemotherapy, median time from last treatment to SBRT was 3 months (range: 1 month to 48 months). Median chemotherapy-free interval prior to SBRT (determined from the time of last chemotherapy or diagnosis, if received no chemotherapy) was 19 months (range: 1 month to 48 months) for all patients. No patients received concurrent chemotherapy with SBRT.

Treatment characteristics are presented in Table 2. Median size of the treated lymph node metastases was 1.95 cm (range: 0.8–6.2 cm). RT doses were 31 to 60 Gy in four to ten fractions, with 44% of patients receiving 35 Gy in 5 fractions. Median V95 coverage of the PTV was 96% (range: 60–100%). Median prescription biological equivalent dose assuming an α/β of 10 (BED_10_) was 59.5 Gy (range: 54.8 to 105 Gy). Median BED_10_ to 95% of the PTV was 56.6 Gy (range: 29.8–72.0 Gy). Four of eighteen patients (22%) received a BED_10_ of >70 Gy to 95% of the PTV, while 12 patients (67%) received a BED_10_ of >50 Gy.

**Table 2:** Treatment characteristics

Characteristics	All Patients (*N* = 18) n (%)
Size of LN metastases, cm (median, (range))	1.95 (0.8, 6.2)
GTV volume, cm^3^ (median, (range))	8.4 (0.7, 67.8)
PTV volumes, cm^3^ (median, (range))	34.8 (6.5, 162.2)
SBRT dose (BED_10_ Equivalent)^a^	
60 Gy/8 (105 Gy)	1 (5.6)
50 Gy/10 (75 Gy)	2 (11.1)
40 Gy/5 (72 Gy)	5 (27.8)
35 Gy/5 (59.5 Gy)	8 (44.4)
31 Gy/4 (55 Gy)	1 (5.6)
33 Gy/5 (54.8 Gy)	1 (5.6)
Prescribed BED_10_, Gy (median, (range))	59.5 (54.8, 105)
D95^b^ BED_10,_ Gy (median, (range))	56.6 (29.8, 72.0)
D95 BED_10_ > 70 Gy	4 (22.2)
D95 BED_10_ > 50 Gy	12 (66.7)
PTV V100, %	
Mean ± SD	78.6 ± 22.4
Median (Range)	90.0 (21.0, 95.2)
PTV V95, %	
Mean ± SD	87.5 ± 14.7
Median (Range)	95.6 (60.4, 100)
PTV V90, %	
Mean ± SD	91.7 ± 10.8
Median (Range)	97.8 (67.6, 100)

^a^BED_10_: Biological Equivalent Dose assuming α/β of 10

^b^D95: Minimum dose to 95% of PTV

### Toxicities and outcomes

There were no grade ≥3 toxicities reported. There were only 3 cases of grade 2 toxicity; all nausea related to treatment in the abdomen. Most common adverse effect for all patients was grade 1 fatigue.

**Fig. 1:**
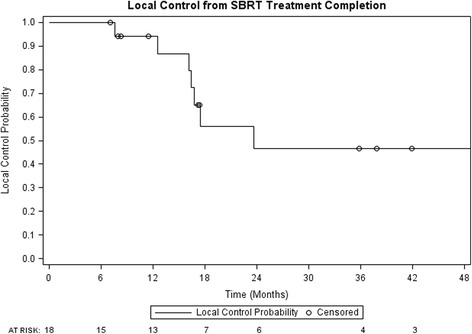
Local control from time of SBRT completion

**Fig. 2:**
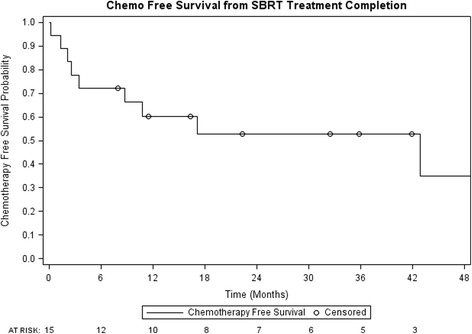
Chemotherapy-free survival from time of SBRT completion

**Fig. 3:**
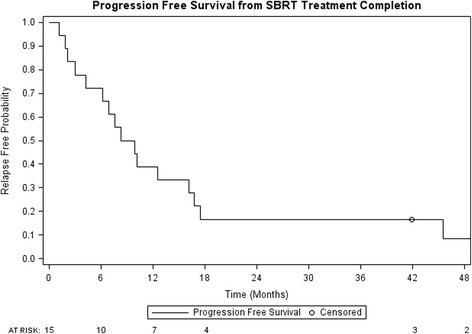
Progression-free survival from time of SBRT completion

**Fig. 4:**
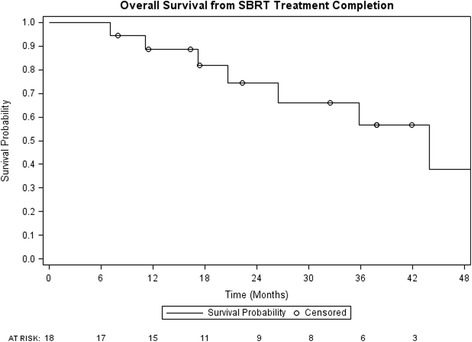
Overall survival from time of SBRT completion

One and 2 year local control were 94% (95% confidence interval (CI): 83–100%) and 47% (95% CI: 18–75%) respectively (Fig. 1). Post SBRT chemotherapy-free survival was 60% (95% CI: 37–83%) and 53% (95% CI: 28–77%) at 1 and 2 years (Fig. 2). PFS at 1 and 2 years were 39% (95% CI: 16–61%) and 17% (95% CI: 0–34%) respectively, while 1 and 2 year OS were 89% (95% CI: 74–100%) and 74% (95% CI: 52–96%) (Figs. 3 and 4, respectively). At the time of this analysis (data frozen on June 10, 2016), two patients were alive and free of disease and seven patients died with the median interval from disease recurrence to death in these 7 patients of 19 months (range: 2–31 months). Four patients presented with failure at the SBRT site and 12 patients presented as distant disease as first site of disease recurrence. All patients with failure at the SBRT site underwent further local treatments including RFA, SBRT and surgery. Two of the four patients subsequently received delayed chemotherapy as part of their treatment. For those who progressed distantly, 7 patients (58%) had chemotherapy as part of their treatment, 3 patients underwent additional SBRT to their distant sites of recurrence to delay systemic therapy while 2 patients received no further therapies.

## Discussion

The incidence of lymph node metastasis after curative primary treatment depends on the tumor histology and primary site, but has been reported as 15–20% [18, 19]. Extrapolating from data showing that achieving an R0 resection of recurrent pelvic disease in colorectal cancer is prognostic for OS, the local ablation of metastatic lymph nodes may alter disease prognosis [20, 21]. Similarly, several studies have shown improved survival rates with complete resection of retroperitoneal, intra- abdominal and para-aortic lymph node recurrences [22, 23]. Unfortunately, surgical resection of oligometastatic lymph nodes remains technically challenging in many cases. Previous therapies to the site of metastases or recurrence may lend to increased risk of surgical morbidity. Furthermore, retroperitoneal locations of many lymph nodes make complete resections difficult to achieve [24]. SBRT offers a strategy for treatment of oligometastatic lymph nodes that are a challenge to resection, as potentially ablative doses of radiotherapy can be delivered with high conformality and a rapid dose drop-off gradient anywhere in the body.

Our 1 year LC rate is excellent at 94% while our 2 year LC rate at 47% compares less favorably than other reported series. In the largest published reported series to date on SBRT treatment of solitary lymph node metastasis, LC at 3 years was 64% [25]. The differences in local control however, may result from the use of concomitant chemotherapy and difference in primary tumors. In the study by Jereczek-Fossa et al., 69 patients were treated with a median dose of 24 Gy in 3 fractions to a single abdominal lymph node recurrence from various tumor primaries. Although a dose of 24 Gy in 3 fractions, equivalent to a BED_10_ of 43.2 Gy, is lower than the median BED_10_ dose of 59.5 Gy in our current study, 37% of lesions in the study by Jereczek –Fossa et al. were treated concomitantly with systemic chemotherapy. As well, approximately half of the patients in the study by Jereczek-Fossa et al. had a prostate or gynecological primary [25]. Therefore, histology, rather than dose may have affected LC rates. In our series, all but one patient had a gastrointestinal primary, and over a third of the patients had a colorectal primary tumor site. Data from other studies of metastatic disease suggest that gastrointestinal primary tumor metastases, particularly colorectal primaries, have worse local control than metastases from other histologies which may account for the lower 2-year LC in our study [26]. Furthermore, the inclusion of five patients with either pancreas or gallbladder cancer, may have reduced our local control.

Our 1 and 2 year OS at 89% and 74% are favorable compared to results from other published data. Jereczek-Fossa et al. reported a 3-year OS of 50% [25]. Milano et al. reported a 2-year OS of 50% in a series of 121 patients with ≤ 5 detectable metastases treated with SBRT. Only 28 patients (23%), however, had lymph node metastasis, with the majority having lung or liver metastases, making comparisons to our current study challenging [15]. In a smaller series by Bignardi et al., 19 patients with unresectable retroperitoneal nodal metastases in the abdomen were treated with SBRT. 1 and 2 year OS was comparable to our study at 93.3% ± 6.4%. All patients were treated with 45 Gy in 6 fractions and 11 of the 19 patients had solitary nodal metastasis at time of treatment [4].

Despite good LC rates after SBRT, PFS in our study was low at 17% at 2 years but is in line with published literature. Jereczek-Fossa et al. reported a 3 year PFS of 12% [25]. Similarly, Milano et al. reported a 2-year PFS of 16% [15]. Most patients in our study progressed outside of the radiotherapy treatment volume, which is in agreement with observations from other investigators [27]. Although some may view a low PFS as a failure of SBRT treatment, a 2-year 17% PFS translates to approximately 1 in 6 patients surviving 2 years without disease recurrence, without need for further chemotherapy and having minimal toxicity from radiation treatment. Nonetheless, the low PFS does highlight the fact that most patients will eventually require systemic therapy. Further investigations are required to identify the optimal sequencing of local ablative therapies, such as SBRT, with systemic therapies.

To our knowledge, no prior studies to date have reported CFS post SBRT as an outcome. Patients with oligometastatic disease to lymph nodes are asymptomatic and generally have a low volume of disease. Although local control to limited metastases may provide cure to only a few well-selected patients, local control may still be clinically relevant to delay the institution of chemotherapy [4]. Delaying chemotherapy may avoid adverse effects associated with systemic therapies, thereby preserving quality of life. Our CFS was 60% at 1 year and 53% at 2 years. Historically, without surgical options, our patients would have been offered upfront chemotherapy as standard of care. Whether or not patients would still been free from chemotherapy at 2 years without SBRT remains unanswered from this study. Interestingly, 7 of the 16 patients who progressed in our study were amenable to upfront locoregional therapies such as surgery, alcohol ablation, Y-90 and SBRT, which further delayed initiation of systemic therapy.

In our series, PTV coverage was good despite often challenging location of lymph nodes near organs at risk such as small bowel or proximal tracheal tree. Our mean PTV V95 coverage was 87.5 ± 14.7% while our median BED_10_ to 95% of the PTV was 56.6 Gy. There were no grade 3 or higher toxicities reported in our cohort which supports the low toxicity profile in SBRT in the treatment of oligometastatic lymph node disease [24]. Our low toxicities may be related to the inherent steep dose-gradient achievable by SBRT, the use of CBCT image guidance prior to treatment delivery as well as our choice of dose and fractionation. Various fractionation schemes have been described in literature, but the optimal dose and fractionation for lymph node metastases is unknown [28]. We used a heterogeneous dose and fractionation scheme common to early SBRT studies due to unknown dose limitations and toxicities [25, 28]. BED_10_ doses have been reported in this study in order to allow for comparison of outcomes to future studies. In light of the low toxicity from SBRT in our study, one may hypothesize that there is room for potential dose escalation.

We are aware that our current study has several limitations including its retrospective nature, small sample size, and heterogeneity in the prescribed dose and tumor histologies. The retrospective nature of this study limited our ability to report and capture results of patients who met all eligibility criteria but were not treated with SBRT. Furthermore, although RECIST criteria was used for evaluating local response to treatment, we were unable to capture scaring or fibrosis from treatment as not all patients underwent PET evaluation in follow-up. Larger retrospective cohorts and prospective clinical studies are likely required to determine the optimal treatment dose as well as to better identify a subset of patients with oligometastatic nodal disease who benefit the most from SBRT.

## Conclusion

In this single institution study, SBRT to oligometastatic lymph nodes provides high local control and a moderate chemotherapy-free interval with acceptable toxicities. Progression of disease remains prominent in these patients, but many may be eligible for further local treatments delaying the institution of systemic therapies. Further investigations to increase cohort patient numbers should be performed to confirm these results and better identify a subset of patients who benefit the most from SBRT.
